# Analysis of health expenditures in China from 2000 to 2019 compared with the world and upper-middle-income countries

**DOI:** 10.3389/fpubh.2024.1464214

**Published:** 2025-02-06

**Authors:** Xindian Zeng, Lijie Chen, Lu Chen

**Affiliations:** ^1^The Affiliated Nanhua Hospital, Hengyang Medical School, University of South China, Hengyang, Hunan, China; ^2^Key Laboratory of Hengyang for Health Hazard Factors Inspection and Quarantine, Hengyang, Hunan, China; ^3^Department of Nephrology, First Medical Center of Chinese People's Liberation Army General Hospital, Nephrology Institute of the Chinese People's Liberation Army, State Key Laboratory of Kidney Diseases, National Clinical Research Center for Kidney Diseases, Beijing Key Laboratory of Kidney Disease Research, Beijing, China

**Keywords:** THE, GHE, SHE, OOPHE, the financing structure

## Abstract

**Background:**

Fairness in health funding has always been a priority in China. This article aims to study the trends in total health expenditure (THE), government health expenditure (GHE), social health expenditure (SHE), and out-of-pocket health expenditure (OOPHE) among China, the world, and upper-middle-income countries from 2000 to 2019. The goal is to provide a theoretical basis for the Chinese government to adjust and formulate health-related policies.

**Methods:**

Aggregate time-series data were collected from the World Health Organization (WHO) and the World Bank (WB) Open Data sources from 2000 to 2019. These data were compared and some of it analyzed using cluster analysis methods.

**Results:**

The financing structure level of THE in China is currently above average among upper-middle-income countries. The proportion of THE in GDP for upper-middle-income countries and China remains relatively stable and slow growth, and is consistently lower than the world average. The proportions of GHE and SHE in THE in China and upper-middle-income countries converged toward the world average level. But the proportion of OOPHE in THE in China is higher than that of two. Overall, the proportions of GHE exhibited an upward trend and the proportion of SHE and OOPHE exhibited a downward trend in China. What's more, the changes in China are more pronounced. The proportion of THE in GDP of China increased by 0.84% from 2000 to 2019, and it ranked 24th among the 51 upper-middle-income countries in 2019, and Compared with it in 2000 there has been a consistently positive increase rate. The per capita health expenditure in China was $42.11 in 2000 and it's $535.13 in 2019, which rapidly grow to the level of upper-middle-income countries and narrow the gap with the level of the world.

**Conclusion:**

The financing structure of THE is increasingly optimized, but the level of financing still needs improvement in China. The government should continue to optimize the financing structure of THE, increase GHE, encourage social capital investment, decrease the proportion of OOPHE, diversify financing and reimbursement policies to promote hierarchical medical system, promote health management for an aging population, and formulate health expenditure plans for public health emergencies.

## 1 Background

Healthcare and national health are closely related to the economy of a country and the wellbeing of its people. In China, medical and health services have always been highly valued by the government. Before 2011, the proportion of OOPHE in China was relatively high, leading to a heavy health burden, especially for rural households in underdeveloped areas (1). The healthcare of residents and public health issues have also received increased attention from the public.

Changes in medical and health expenditure not only reflect the economic development of China but also indicate shifts in the health mindset of the Chinese people, influencing the improvement of the Chinese social security system. THE refers to the total amount of money spent by society on health services in a region or country during a certain period (2). It comprises GHE, SHE, and OOPHE.

THE can reflect the relationship between health policies and economic development and provide information for the government to formulate health strategies. SHE consists mainly of two parts: the basic medical insurance fund and social capital investment paid by units and individuals (3). The proportion of OOPHE is positively correlated with underdevelopment and social injustice, reflecting the medical burden on individuals (3, 4). The percentage of GHE in THE reflects the importance and investment of government departments in healthcare in a country or region. Health status is not only related to the total amount of THE but also to the structure among the three components (5). The proportion of THE in GDP (gross domestic product) reflects the level of financial investment in healthcare by different countries or regions at different stages of economic development. It is also commonly used to study whether the health expenditure of different countries or regions are in line with the local economic development and the health service needs of residents (6–10).

This paper uses comparative analysis to study the relevant data of THE, reflecting the level, structure, and changing trends of health expenditures, and explores the underlying laws. Studying the changes in the proportion of GHE, SHE, and OOPHE have positive significance in solving the problems of difficulty in accessing medical care, reducing heavy medical burden, optimizing the allocation of health resources, improving the accessibility of health services, and promoting national health. Few articles not only analyzed the changes in health expenditure and the three indicators but also explored the comparison of health expenditure in China, the world and upper-middle-income countries. Furthermore, it studied in a time span up to 20 years. So, this article discussed the current changes in health expenditure in China compared with the world and upper-middle-income countries, analyzed the characteristics of these changes in China, and identified the causes of these changes. It further explains the significance of policy formulation and theoretical support for health expenditure. It also tells us what lessons can be learned. This comparative analysis is more convincing.

## 2 Materials and methods

### 2.1 Materials

The data was sourced from the official websites of the World Health Organization (WHO) and the World Bank. According to the latest income grouping criteria released by the World Bank in 2012, upper-middle-income countries are defined as countries with the Per Capita GNP ($4,086–$12,615). In 2013, the Per Capita GNP was $6,740, which has entered the ranks of upper-middle-income countries (11, 12).

### 2.2 Methods

#### 2.2.1 Comparative analysis

Comparative analysis was used to compare and analyze related indicators of THE in China, the world, and upper-middle-income countries.

#### 2.2.2 Cluster analysis

Cluster analysis was conducted by SPSS statistical software on three variables: the proportion of GHE in THE, the proportion of SHE in THE, and the proportion of OOPHE in THE in upper-middle-income countries. Based on the clustering results, differences in the structure and amount of health financing among upper-middle-income countries were compared.

## 3 Result

### 3.1 Cluster analysis of the components of the THE between China and upper-middle-income countries

Based on the health expenditure data provided by the world Health Organization for 51 upper-middle-income countries (a total of 54 upper-middle-income countries, with missing data for American Samoa, Libya, and Kosovo). Fifty countries (except China) are divided into four categories based on health investment (Table 1). Low-input countries (Cluster I) included seven countries such as Tuvalu, Botswana etc. Low-middle-input countries (Cluster II) included 23 countries such as Palau, Fiji, Colombia etc. Upper-middle-input countries (Cluster III) included 16 countries such as Moldova, the Russian Federation, Serbia etc. High-input countries (Cluster IV), included 4 countries such as Azerbaijan, Equatorial Guinea etc. The proportion of GHE in THE, Cluster I of it is the lowest (17.22%), increasing gradually in each category, and Cluster IV of it has the highest proportion (74.70%). While China accounts for 55.98%, which is between Cluster II and Cluster III, leaning toward Cluster III. The proportion of SHE in THE, Cluster I of it is the lowest (14.14%), increasing gradually in each category, and Cluster IV of it has the highest proportion (78.58%). While China accounts for 44.02%, which is between Cluster II and Cluster III, leaning toward Cluster III. The proportion of OOPHE in THE, Cluster I of it is the lowest (11.31%), increasing gradually in each category, and Cluster IV of it has the highest proportion (76.25%). While China accounts for 35.23%, which is between Cluster II and Cluster III, leaning toward Cluster II. In summary, the financing structure level of THE in China is currently above average among upper-middle-income countries.

**Table 1:** Cluster analysis of the components of the THE between China and upper-middle-income countries.

**Region**	**Proportion of GHE in THE (%)**	**Proportion of SHE in THE (%)**	**Proportion of OOPHE in THE (%)**
China	55.98	44.02	35.23
Cluster I	17.22	14.14	11.31
Cluster II	44.30	34.17	29.98
Cluster III	61.02	52.29	45.26
Cluster IV	74.70	78.58	76.25

### 3.2 Comparative analysis of the changing trends of the proportion of THE in GDP and the proportion of GHE, SHE, and OOPHE in THE for China, the world, and upper-middle-income countries from 2000 to 2019

**Figure 1:**
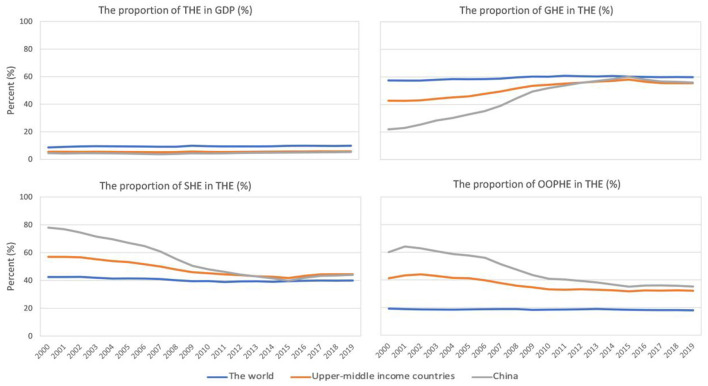
The changing trends of the proportion of THE in GDP and the proportion of GHE, SHE, and OOPHE in THE in China, the world and upper-middle-income countries from 2000 to 2019.

The trend shows that the proportion of THE in GDP for upper-middle-income countries and China remains relatively stable and slow growth, and is consistently lower than the world average (Figure 1). Regarding the proportion of GHE in THE, China has consistently had a higher proportion since 2012 compared with upper-middle-income countries and it reached a peak of 60.18% in 2015, then the maximum gap is 20.79% in 2000 and the minimum gap is 0.05% in 2012 compared with upper-middle-income countries. Regarding the proportion of SHE in THE in China, it is lowest (39.81%) in 2015 and it is lower than upper-middle-income countries since 2014. The proportion of OOPHE in THE in China, it is lowest (35.23%) in 2019 and is highest (64.19%), but it is higher than upper-middle-income countries and the world from 2000 to 2019. So the proportions of GHE and SHE in THE in China and upper-middle-income countries converged toward the world average level. But the proportion of OOPHE in THE in China is higher than that of two. Overall, the proportion of GHE exhibited an upward trend and the proportion of SHE and OOPHE exhibited a downward trend in China. What's more, the changes in China are more pronounced.

### 3.3 Changes in the proportion of THE in GDP from 2000 to 2019 in upper-middle-income countries

**Figure 2:**
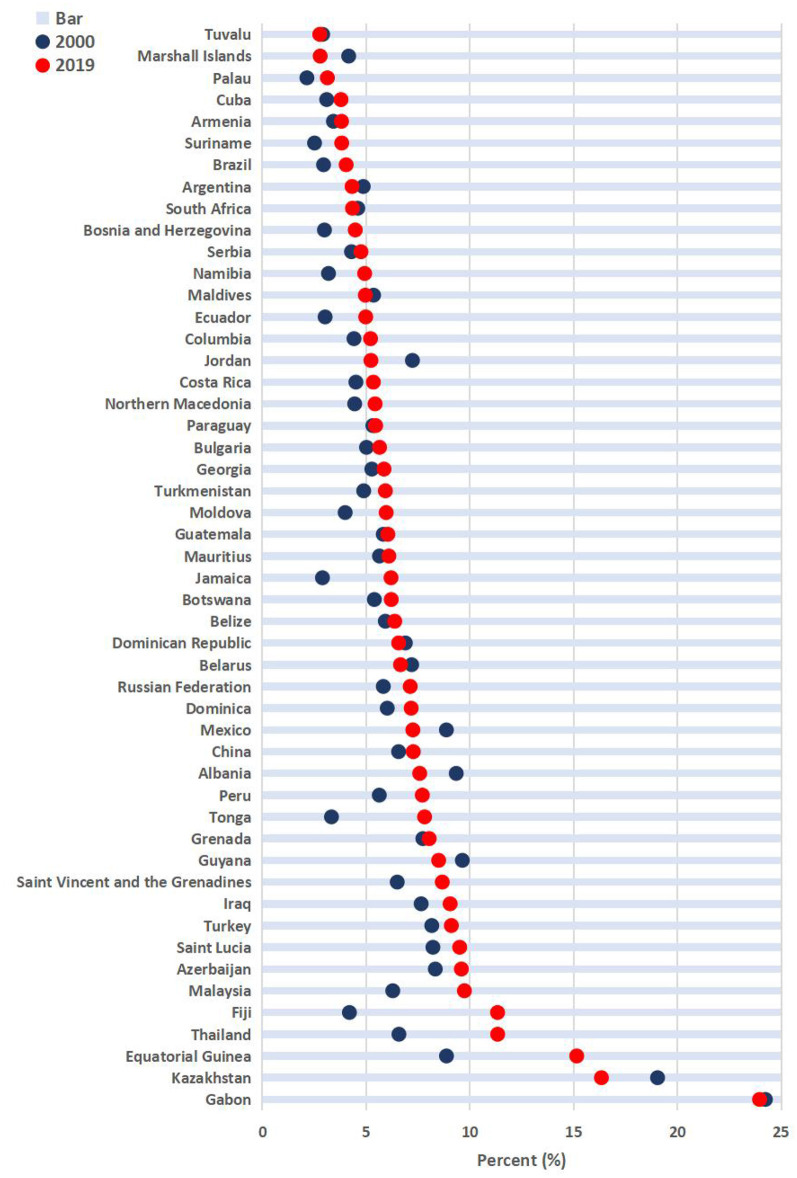
The changes in the proportion of THE in GDP in upper-middle-income countries from 2000 to 2019.

Figure 2 illustrates the changes in the proportion of THE in GDP from 2000 to 2019 in upper-middle-income countries. The data shows that among the 51 countries analyzed, the proportion of THE in GDP for 13 countries (including Gabon, Kazakhstan, Saint Lucia, Turkey, Grenada, Albania, Turkmenistan, Georgia, North Macedonia, Jordan, Namibia, Marshall Islands, and Tuvalu) slightly decreased, while the remaining countries showed an overall upward trend. Among upper-middle-income countries, Armenia had the fastest growth rate in the proportion of THE in GDP from 2000 to 2019, followed by Palau. Marshall Islands experienced the most rapid decline, followed by Albania.

Armenia experienced the fastest upward trend (7.14 percentage points), while Marshall Islands had the fastest downward trend (2.70 percentage points). Overall, from 2000 to 2019, the proportion of THE in GDP of China increased by 0.84%, which was on average among upper-middle-income countries. In 2019, the proportion of THE in GDP in China (5.35%) ranked 24th among the 51 upper-middle-income countries.

### 3.4 Comparative analysis of per capita health expenditure and its increase rate in China, the world, and upper-middle-income countries from 2000 to 2019

Table 2 presents the per capita health expenditure and its increase rate in China, the world, and upper-middle-income countries from 2000 to 2019. The data shows that per capita health expenditure in China has been steadily increasing, with the fastest growth rate observed from 2007 to 2008. However, the rate of increase has been gradually slowing down since then. Compared with the world and upper-middle-income countries, the per capita health expenditure of China has shown relatively rapid growth. For instance, the cycle-increase rate in China was 35.86% in 2008, significantly higher than the rate of 12.30% in the world and the rate of 21.86% in upper-middle-income countries.

**Table 2:** The per capita health expenditure in China, upper-middle income countries and the world, 2000–2019.

**Year**	**China**		**Upper-middle-income countries**		**The world**	
	**Per capita health expenditure (US$)**	**Cycle-increase rate (%)**	**Per capita health expenditure (US$)**	**Cycle-increase rate (%)**	**Per capita health expenditure (US$)**	**Cycle-increase rate (%)**
2000	42.11	…	108.99	…	478.81	…
2001	43.66	3.68	108.46	−0.49	491.89	2.73
2002	49.14	12.55	106.32	−1.97	522.68	6.26
2003	55.65	13.25	118.79	11.73	586.99	12.30
2004	63.26	13.67	138.92	16.95	646.49	10.14
2005	71.86	13.59	165.13	18.87	685.83	6.09
2006	81.23	13.04	192.21	16.40	728.20	6.18
2007	97.08	19.51	232.51	20.97	797.74	9.55
2008	131.89	35.86	283.33	21.86	864.02	8.31
2009	162.62	23.30	292.31	3.17	874.50	1.21
2010	186.49	14.68	338.04	15.64	911.89	4.28
2011	236.37	26.75	397.82	17.68	986.35	8.17
2012	281.68	19.17	428.29	7.66	995.70	0.95
2013	326.04	15.75	464.87	8.54	1,011.83	1.62
2014	359.31	10.20	480.05	3.27	1,034.95	2.28
2015	390.10	8.57	456.86	−4.83	993.98	−3.96
2016	395.36	1.35	447.42	−2.07	1,015.91	2.21
2017	437.26	10.60	503.59	12.55	1,056.65	4.01
2018	501.06	14.59	531.72	5.59	1,103.48	4.43
2019	535.13	6.80	551.87	3.79	1,121.97	1.68

US$, United states dollars.

There has been a consistently positive increase rate over the past 20 years, peaking from 2007 to 2011 before gradually slowing down. In 2000, the per capita health expenditure in China was $42.11, approximately 1/3 of the expenditure in upper-middle-income countries ($108.99) and about 1/11 of that in the world ($478.81). By 2019, the per capita health expenditure in the world had reached $1,121.97 and that of upper-middle-income countries was $551.87, which were respectively 2.10 times and 1.03 times higher than it of China ($535.13).

## 4 Discussion

The analysis of indicators related to THE in China, the world, and upper-middle-income countries from 2000 to 2019 indicates that China has the rapid development in health industry over the past two decades. The THE, as an important indicator reflecting the importance of a country or region to the healthcare and the health of its residents, has become a widespread focus of attention for the people. THE continues to grow, the proportion of GHE in THE is increasing, and the proportions of SHE and OOPHE in THE are decreasing, indicating a continuously optimizing financing structure, which is consistent with the research results of Chai and Zhai (13, 14).

From the results of the cluster analysis in Table 1, it can be seen that the proportion of GHE and SHE in China are both lower than that of Cluster III, and there is still a certain gap with Cluster IV. The proportion of OOPHE in China is leaning toward Cluster II, accounting for 35.23%. So compared with the high level of upper-middle-income countries, there is still much room for improvement in GHE, SHE, and OOPHE. Of course, economic growth remains an important factor influencing the growth of health expenditure in China. Firstly, over the past 20 years, with the economy of China rapidly developed, the demand for health services from residents has increased and China can also afford higher health expenditure than before, resulting in a rapid increase in health expenditure. Secondly, the population aging of China is getting more and more serious. With the increase of the older adult population, the increase in demand for medical services will further need much health expenditure. Finally, this is mainly because China issued policies of the new healthcare system reform in 2009 to deepen the reform of the medical and health system. That makes more people have the access to cure their diseases. These are the reasons that have led to significant changes of health expenditure in China and that's also why the proportion of GHE in THE it reached a peak of 60.18% in 2015, then the maximum gap is 20.79% in 2000 and the minimum gap is 0.05% in 2012 compared with upper-middle-income countries. But the growth rate has slowed down in recent years, which suggests that the growth of government health investment faces the problems of sustainability and stability. The proportions of GHE and SHE in THE in China and upper-middle-income countries converged toward the world average level. But the proportion of OOPHE in THE in China is higher than that of two, the lowest is still 35.23% in 2019. What's more, the changes of three indicators in China are more pronounced. It also verified that the new healthcare system reform has played a positive role. Although the proportion of OOPHE in China was 35.23% in 2019, it is still higher than the international experience limit for eliminating catastrophic family health expenditure (20%) (15). Overall, the proportion of GHE exhibited an upward trend and the proportion of SHE and OOPHE exhibited a downward trend in China, which reflected that the financing structure of THE is increasingly optimized. The proportion of THE in GDP of China increased by 0.84% from 2000 to 2019. The per capita health expenditure is not only an important indicator for measuring the medical and health investment of a country or region, but also a key data for evaluating the effectiveness of medical security policies, ensuring the health status of residents, and optimizing resource allocation. It has been a consistently positive increase rate in China over the past 20 years, approaching the level of middle to upper-middle-income countries. Although the overall trend of it is growing, it is significantly lower than the average level of the world. Before the new healthcare system reform, the main influencing factors of health expenditure in China were demographic structure characteristics, education level, and the health status of the population. It is precisely because of the changes in the proportion of the aging population (65 years old and above) in the total population, the proportion of education expenses in GDP, and the average life expectancy that the demand for health has rapidly increased with economic development. After the new healthcare system reform, the emphasis on the medical security system in China has increased, and investment has continued to increase. The main part of GHE is medical security expenditure of government for new rural cooperative medical system, basic medical security for urban residents, medical assistance systems in urban and rural areas, etc. The effectiveness of Chinese health reform in recent years is evident (16), with a health financing structure level above the middle level of upper-middle-income countries. However, the level of health expenditure in China currently has consistently been lower than the average level of the world. This suggests that further efforts can be made in the following aspects.

The financing structure of health expenditure in China needs further optimization. Firstly, we can strengthen national macroeconomic control. Increase GHE and encourage social investment. The reform of the medical and health system in China needs further promotion. From these results we know the changes are relatively large in China. This reflects ongoing challenges in government health investment and suggests a need to focus on population health in it. Secondly, we should optimize the financing structure of government health investment. Promote new drug research, advanced medical equipment production, and other aspects. Government health expenditures have a positive impact on the efficiency of the medical and health system. Thirdly, the government should reduce OOPHE. Precise measures are needed to reduce the burden of OOPHE and promote the high-quality development of health services. In the relevant health policies of Chinese “Healthy China 2030” planning, one of the goals is that the proportion of OOPHE in THE in China will decrease to 25% in 2030, thereby reducing the proportion of the disaster expenditure of family and protecting from the risk of health financing (17). Finally, we should encourage social capital investment. With the development of the market economy in China and the expansion of social capital, the government should strengthen supervision and encourage social capital to invest in medical institutions. Cooperation between the government and social institutions, along with an increase in investment rates, can have a positive impact on improving medical conditions and the level of medical services.

The continuous increase in THE in China will exert significant pressure on society, families, and individuals. Therefore, it is crucial to establish and improve a diversified medical security system, especially considering the differences in health expenditures among different regions of China (8). The government should diversify financing and reimbursement policies to promote hierarchical medical system (18). The government also should promote commercial medical insurance to weaken income restrictions on timely access to medical services for residents (19). We can provide preferential policies to commercial medical insurance companies and offer policy support and personal tax reductions for residents who purchase commercial medical insurance. This can strengthen disease prevention and treatment, thereby improving health quality of residents (20). At present, DRG (Diagnosis Related Group) and DIP (Diagnosis-Intervention Packet), two payment methods for medical insurance, have been piloted and operated nationwide, accelerating the complete transformation of payment models. The result of practice has shown that both payment methods have reduced the economic burden on patients with a certain extent, reduced OOPHE, and have a positive effect on medical reform. This reforms have effectively promoted the high-quality and efficient development of public hospitals to assist in achieving win-win results for the government, public hospitals and patients (21, 22). Firstly, it is necessary to strengthen the understanding and training of medical staff on the significance, objective and content of clinical pathways and improve their attention on DRG and DIP, because they are the main participants in the promotion of this model (21–23). Secondly, it is suggested to achieve scientific cost control through continuous improvement of clinical pathway management. At the same time, ensuring the quality of medical care is equally important (24). Thirdly, compared to the fee-for-service (FFS) payment, the prices of drugs and materials with DRG and DIP payment have significantly decreased (25, 26). We can actively promote transformation of medical insurance payment methods. China can promote the transformation from the FFS payment system to the DRG or DIP case-mix payment system.

The government should strengthen health management of the aging population. The issue of it in China is quite prominent. China has entered an aging society since the end of 1999, and the population of older adult people (aged 65 years and above) in China has exceeded 200 million, ranking first in the world by the end of 2021 (27). According to the World Population Prospects 2019 released by world Nations, the aging level of China will continue to deepen, reaching 26.1% by 2050 and 30.1% by 2075 (28, 29). The population aging of China have the characteristics of large scale, with rapid growth rate, obvious regional differences, and aging before being rich. With the increasing proportion of the population of older adult people (aged 65 years and above) in China, the degree of aging in the country is deepening, and the prevalence of older adult is constantly increasing. As a result, the demand of older adult for medical and health services has increased, and more health resources need to be invested (23). In addition, changes in the disease spectrum and the shift in the focus of attention of residents will affect the health funding structure. Of course, it is imperative to take measures to reduce the risk factors of major non-communicable disease (NCDs), increase public health funding and raise the overall benefit packages for social health insurance schemes, and ensure healthy aging (30). Reinforce health education to improve public awareness, particularly among the older adult, who often neglect preventive measures and only seek medical treatment when symptoms are severe. Establish multi-level health education and guidance at the national, social, family, and individual levels to promote healthy aging. Long-Term care system and health insurance scheme are made. Develop a long-term care system and related health insurance schemes to address the changing disease spectrum and focus of attention of residents, ensuring healthy aging.

The government should formulate health expenditure plans in response to public health emergencies. After the SARS outbreak, China began to reform its public health infrastructure, leading to a significant increase in health expenditure (31). However, the outbreak of COVID-19 highlighted a shortage of health resources, prompting the government to swiftly scale up epidemic emergency capacity and managing public health emergencies effectively (32). Despite these efforts, there was no specific health expenditure plan for public health emergencies, which slowed down the speed of epidemic response. The expenditure of medical expenses is unique, affected not only by social, economic, and policy factors but also by public health emergencies. This results in non-linear and unstable time series of medical expenses, as seen in the substantial increase in Chinese public health expenditure after the COVID-19 outbreak (33). However, several challenges remain. Chinese public health expenditure is significantly lower than that of developed countries. Public health resources are not well-coordinated, leading to an imbalance and inequity in public-health resource allocation among provinces (34). To optimize total health expenditure, a health expenditure plan should be developed for public health emergencies. This plan should include that optimizing the emergency early warning system of government for public health emergencies and accelerating the construction of an emergency medical reserve base.

## 5 Conclusion

The financing structure of THE is increasingly optimized in China, but the level of financing still needs improvement. The government should continue to optimize the financing structure of THE, increase GHE, encourage social capital investment, reduce OOPHE, diversify financing and reimbursement policies to promote hierarchical medical system, promote the health management of the aging population, and formulate health expenditure plans for public health emergencies.

## Data Availability

Publicly available datasets were analyzed in this study. This data can be found here: the data was sourced from the official websites of the World Health Organization (WHO) and the World Bank.
